# The archerfish predictive C-start

**DOI:** 10.1007/s00359-023-01658-2

**Published:** 2023-07-23

**Authors:** Stefan Schuster

**Affiliations:** Lehrstuhl für Tierphysiologie , University of Bayreuth , 95440 Bayreuth, Germany

**Keywords:** Decision-making, Neuroethology, C-start, Predator, Speed–accuracy

## Abstract

A very quick decision enables hunting archerfish to secure downed prey even when they are heavily outnumbered by competing other surface-feeding fish. Based exclusively on information that is taken briefly after the onset of prey motion, the fish select a rapid C-start that turns them right towards the later point of catch. Moreover, the C-start, and not later fin strokes, already lends the fish the speed needed to arrive at just the right time. The archerfish predictive C-starts are kinematically not distinguishable from escape C-starts made by the same individual and are among the fastest C-starts known in teleost fish. The start decisions allow the fish—for ballistically falling prey—to respond accurately to any combination of the initial variables of prey movement and for any position and orientation of the responding fish. The start decisions do not show a speed–accuracy tradeoff and their accuracy is buffered against substantial changes of environmental parameters. Here, I introduce key aspects of this high-speed decision that combines speed, complexity, and precision in an unusual way.

## A necessary addition to the shooting behavior of archerfish

Archerfish are renowned for their unique hunting behavior. By firing water jets, these fish manage to dislodge unsuspecting aerial prey from overhanging twigs or leaves to then catch it, once it lands on the water surface (e.g., Smith 1936; Lüling 1963; Dill 1977; Sillar et al. 2016). Although shooting water may appear a rather simple thing to do, transferring sufficient force to targets of various sizes in distances ranging from two to twenty times the animal’s own length is not. In fact, archerfish are the only of about 35.000 species of fish that can do this, and no other fascinating spitting creature in the animal kingdom—from spitting spiders to cobras—known so far needs to transfer force to distant targets, but rather must transfer enough glue or venom (reviewed in Schuster 2018). To overcome the adhesive forces of its various prey, the fish need considerable control over the hydrodynamic properties of their water jets, the amount and time course of water release, and need to compensate recoil during the formation of their jets (Schlegel et al. 2006; Gerullis and Schuster 2014; Gerullis et al. 2021). Their ability to hunt distant aerial prey has enabled archerfish to evolve a remarkable combination of fascinating capabilities, from spotting prey in complete absence of any motion cues to efficient ways of learning how to engage it (Schuster 2018).

Given all these capabilities, archerfish should be the unmatched champions in their Southeast-Asian mangrove biotopes. However, a closer look at the conditions that archerfish face in the wild makes one wonder why archerfish shoot at all. Typically, they are accompanied by more numerous other surface-feeding fish, mostly halfbeaks, that would also eat just everything that archerfish down (Rischawy et al. 2015). Because there are so many halfbeaks, it is most likely that one of them is closer to the landing point of prey and so most of all masterfully dislodged prey would go to a halfbeak, not to the shooter or any archerfish bystander. Halfbeaks would not only typically be closer to the landing point of prey, but they also are better at detecting water surface waves (Rischawy et al. 2015). For archerfish, it is, thus, no option to guide their approach to prey using mechanosensory cues generated by the splashing impact of their prey. Instead, they need a much faster solution that does not rely on the comparatively slow propagation of capillary surface waves (Bleckmann 1994) but that informs the fish much earlier where it needs to be and when to make the catch. Archerfish use vision to do this and decide on an appropriate action based on a very quick estimate of the initial movement of falling prey.

**Fig. 1:**
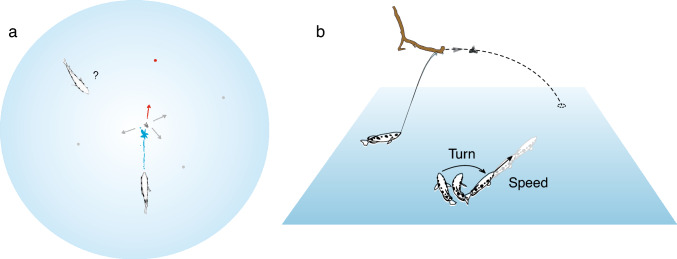
The predictive start decision. Based on the initial movement of falling prey, archerfish decide on a motor pattern that makes them arrive at the right time at the spot where the insect hits the water surface. **a**, **b** Hunting scene as seen from above **a** and from the side **b**. A second archerfish (bystander) is shown besides the shooter. **a** The large blue circle illustrates the area in which the prey could potentially land. A few landing points are shown as gray dots together with possible initial trajectories (gray). The actual landing point can only be inferred once prey has started to fall (red). **b** illustrates, for the bystander, that the starts are C-starts that turn the fish to the later landing point of prey and lend it the speed needed to cover the distance within the remaining time so that the fish will arrive simultaneously with its prey. Adapted from Schlegel and Schuster (2008) and Krupczynski and Schuster (2013)

Figure 1a, b illustrates the decision that archerfish must make. In the depicted hunting scene, one fish shoots, another bystander archerfish just watches what happens. While the shot travels towards its victim neither the shooter nor the bystander know where prey is later going to land on the water surface. This is because, depending on the impact of the water jet, prey can go off in different directions and with different values of speed. Correspondingly, many impact points would be possible. In Fig. 1a, the large blue circle shows where possible impact points could be in a situation where prey falls from 30 cm above the water surface after being dislodged from the lower side of a rigid substrate. With so many possibilities, the fish need to wait until prey is dislodged and starts its ballistic path towards the water surface. When this happens, the fish has only very limited time till impact. During this time, the fish very quickly decides on a motor behavior that, once executed, makes the fish arrive at the landing point just at the time when prey also gets there. The motor behavior that archerfish select in this context is a so-called C-start (see below), at the end of which the fish is not only rotated right to where prey is later going to land but also is propelled with just the right amount of linear speed that is needed to arrive simultaneously with its prey (Fig. 1b).

## The archerfish predictive starts are among the fastest C-starts known in fish

Unlike terrestrial animals, fish obviously cannot accelerate by pushing off against a rigid substrate. In their C-start maneuvers, fish manage to accelerate by first bending their body into the shape of a letter ‘C’ and then pushing water backwards in a subsequent rapid straightening phase (e.g., Weihs 1973; Domenici and Blake 1997; Sillar et al. 2016). Most fish produce such C-starts to escape from sudden danger, such as a rapidly approaching predator. In an escape context, C-starts should be fast, but need not be precise. Rather, an escape direction should be sufficiently variable to not allow the predator to predict the escape trajectory of its prey. In fact, the C-starts of fish are known mostly for high acceleration but not for directional precision or the fine-tuning of the speed they finally lend an escaping fish. Nevertheless, the archerfish predictive starts are not only typical C-starts, but they even are among the fastest C-starts known in teleost fish (Wöhl and Schuster 2007). This is surprising because of the precision these starts need to have over the full range of possible trajectories. It is also worth noting that the archerfish predictive C-starts are performed right at the water surface (Hertel 1966; Webb et al. 1991), so that substantial energy is lost to the production of surface water waves, a problem that most other C-starts examined in the literature (e.g., Domenici and Blake 1997) did not have. Yet, the linear and angular acceleration achieved in the archerfish predictive C-starts is impressive (Wöhl and Schuster 2007). Their top linear acceleration of 120 ms^− 2^ is perhaps matched only by pike (Harper and Blake 1991) whose maximum linear speed, however, remains below 11 body lengths (BL) per second, less than half the archerfish’s maximum of 24 BL/s. Archerfish also reach much higher values in maximum angular speed (up to 5000 deg/s) and angular acceleration (up to 450 000 deg s^− 2^, ten times the angular acceleration of a saccade of a *Drosophila* fly).

**Fig. 2:**
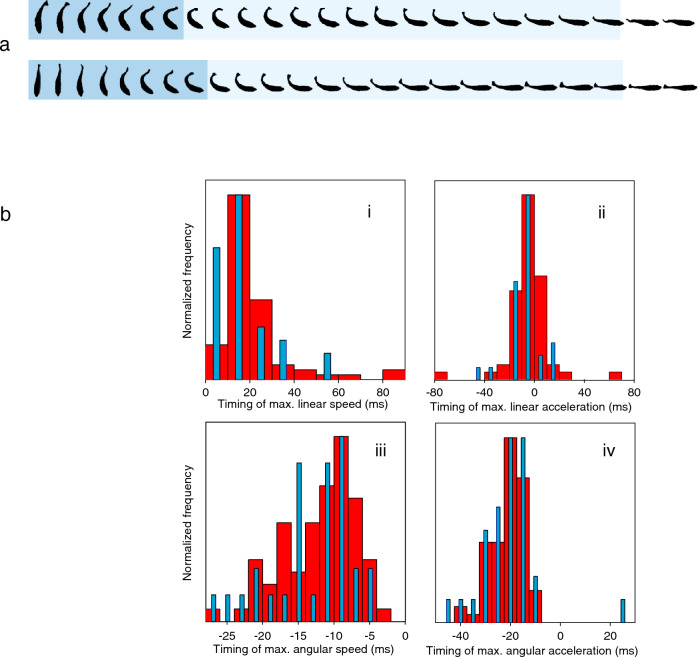
The archerfish predictive start is a top-power C-start that is kinematically not distinguishable from archerfish escape C-starts. **a** Example of an archerfish predictive (upper row) and an escape (lower row) C-start imaged at 500 frames per second (every second frame shown). Background color shows initial bending into the shape of the letter ‘C’ (dark blue; end defined by minimum distance between head and tail) and subsequent straightening phase (light blue) of the C-starts. **b** Example of an analysis showing same temporal characteristics in escape and in predictive C-starts. Timing is reported here relative to the onset of the straightening phase and shows the distribution of when maximal linear speed (i) or linear acceleration (ii) was reached, and when angular speed (iii) and angular acceleration (iv) were maximal. No difference was found between the distributions for escape (blue columns) and predictive (red columns) C-starts. Adapted from Wöhl and Schuster (2007)

Even though they are powerful C-starts, the archerfish predictive C-starts could still be slower than their escape C-starts because of the much higher demand on precision. However, when escape C-starts and predictive C-starts were recorded in the same archerfish individuals, no difference in kinematics could be found. It was impossible, based on the recordings, to tell which C-start was an escape and which one was a predictive start. Both are just full-power C-starts that follow the same kinematics (Fig. 2a, b). The latency of the visually induced predictive C-starts is impressive: Depending on visual contrast and temperature, they can be initiated in as little as 40 ms after onset of prey movement. Once the straightening (so-called stage 2) phase of the C-start is finished, translational speed picked up during the maneuver remains constant for at least 60 ms (Wöhl and Schuster 2006; Krupczynski and Schuster 2013; Reinel and Schuster 2014) and this initial speed of the responding fish is determined by the C-start itself and not by later fin strokes (Reinel and Schuster 2014). Moreover, when the average aim and speed are determined right at the end of the straightening phase, that is, before the fish actually starts its approach path, then it turns out that both are already set just as required: toward the later impact point and so that the fish bridges the distance to this point in the time that is left till impact. Moving at constant speed and arriving just in time, as prepared by the predictive starts, is energetically the best option and less costly than arriving in time but having to accelerate or than swimming too fast and arriving too early (Wöhl and Schuster 2006). The precision of the turn is about 6 deg, i.e., the angle the minute hand of a watch covers in one minute. The large range of possible turns and speed levels and the precision in setting them does not come at all at the cost of a detectable decrease in performance compared to the archerfish escapes and compared to the escape C-starts of other fish.

## An arms-race with competitors

**Fig. 3:**
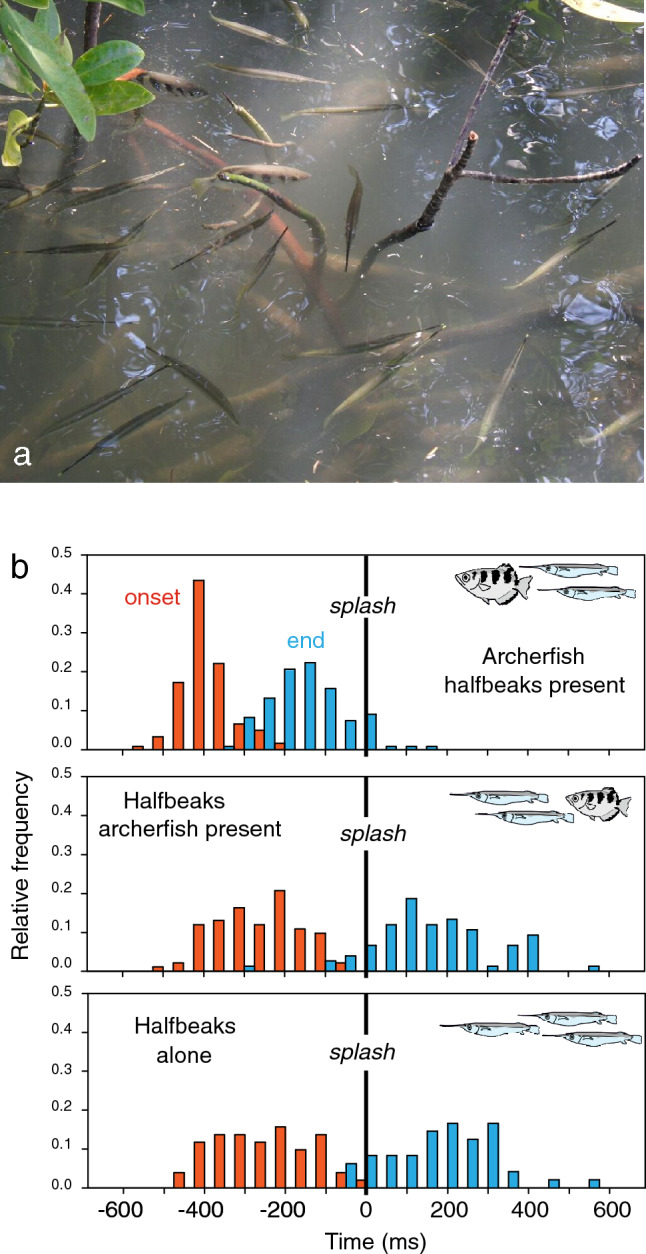
Competition between archerfish and halfbeaks. **a** In Thailand, archerfish (here *Toxotes jaculatrix*) are typically found together with numerous halfbeaks (*Zenarchopterus buffonis*) that eat what archerfish also eat, are active day and night, and are more sensitive to water surface waves. **b** Experiments in the field show that in response to falling prey, archerfish quickly initiate their predictive C-starts and secure over 98% of prey. In these experiments, prey is snatched from a bridge and the responses of the fish below are recorded. In each experiment, the time of onset (red) and end (blue) of the C-start of the first fish of each species was measured. The resulting frequency distribution of C-start onset and end times was determined from 122 experiments. Time is measured relative impact (‘splash’) of ballistically falling prey. Upper: All archerfish C-starts were initiated before prey impact and fish were already under way before impact. Middle: Not only the archerfish but also the competing halfbeaks can initiate responses before the impact of food. Lower: Same timing of the halfbeak pre-impact responses in the absence of archerfish shows that the responses were driven by movement cues from the falling prey and not by archerfish C-starts. Adapted from Rischawy et al. (2015)

Before looking at how the C-start decisions are made, one question is perhaps more important at this time: what do archerfish really gain from their predictive C-starts? Do these starts work well enough under the conditions in the wild to help archerfish get at least some of their downed prey? To determine success rate directly in the field—in the presence of many halfbeaks (Fig. 3a)—pieces of bread were snatched from a platform above the water surface and the responses of the archerfish and halfbeaks below were recorded (Rischawy et al. 2015). These experiments showed that their predictive C-starts helped archerfish to about 98% of catches in the presence of at least ten times more halfbeaks. Given the numerical superiority of the halfbeaks, this success rate is quite impressive, but it is only possible because of the speed and precision of the archerfish starts. This is because also the halfbeaks do not wait till the impact of prey but at least some of them also respond before prey impact and based on vision. The findings, obtained in many tests with different speed and direction of the falling food, are shown in Fig. 3b in which the red columns show when a C-start was initiated and the blue ones when it had just ended, and the fish were on their way. All archerfish in a scene produced their predictive C-starts but some halfbeaks also responded before the impact of food. Their responses were initiated later than those of archerfish and finished only after impact, but they were equally shown without any archerfish present and, thus, driven by visual information from the aerial trajectory of prey. Furthermore, these visually driven starts are also aligned approximately to the later impact point. So, not only archerfish, but also halfbeaks are able to use visual cues to select appropriate turns (Rischawy et al. 2015). Halfbeaks are not the only fish that are similar to archerfish in their ability to analyze aerial motion. For instance, the adults of the fruit-eating riverine ‘machaca’ fish (*Brycon guatemalensis*) of Costa Rica often assemble under fig trees when these release all their fruit within a few days. These fish analyze aerial visual movement to select an appropriate drift-corrected approach path to where a falling fig will later land (Krupczynski and Schuster 2008). So, it is perhaps not too surprising that halfbeaks and probably many more surface-feeding fish share at least partly some aspects of the archerfish’s start decisions. However, the performance of the halfbeaks makes things worse for the archerfish: their predictive starts need to be even faster and more accurate than the starts of their competitors.

The importance of the predictive C-starts can directly be seen in the wild: As soon as it darkens, the advantage that their visually guided predictive C-starts lends to archerfish vanishes, and when this is the case, archerfish stop engaging in any hunting activity while the halfbeaks remain active. What is striking about this is that in the lab, archerfish can still accurately shoot at much lower light levels, even ones at which it is impossible for the dark-adapted human eye to see anything. However, at the light levels at which they stop hunting in the wild, archerfish in the lab can no longer produce their predictive C-starts and it takes them seconds to eventually arrive at where downed prey has landed. The halfbeaks, in contrast, arrive much earlier, about 180 ms after the impact of prey (Rischawy et al. 2015). They, too, can no longer respond visually, but they can still quickly reach downed prey, perhaps because they have more, larger, and more regularly spaced dorsal superficial neuromasts compared to archerfish (Rischawy et al. 2015). Losing the advantage of their predictive starts in the dark means that archerfish would lose most of their downed prey to the halfbeaks and so it is best to stop hunting aerial prey.

The predictive starts require competition even when no halfbeaks are around. When the individuals of a group of archerfish were separated, each individual now in its own tank, then all individual fish still downed insects from a fixed height above the water surface but after some time they no longer showed predictive C-starts. So, the falling motion of prey was still visible and present but now failed to elicit C-starts. However, the starts came back when all individuals were later assembled again in one tank (Schlegel and Schuster 2008). This experiment suggests that competition of any sort is fundamental for the occurrence of the predictive C-starts. It also shows another interesting aspect: As fast and reflex-like as the predictive starts are, they are clearly not ‘reflexively’ elicited anytime something falls towards the water surface: They vanish after prolonged absence of any competitors, and they are not shown when the fish are not hungry.

## All information used in the decision is sampled in a brief interval after motion onset of prey

**Fig. 4:**
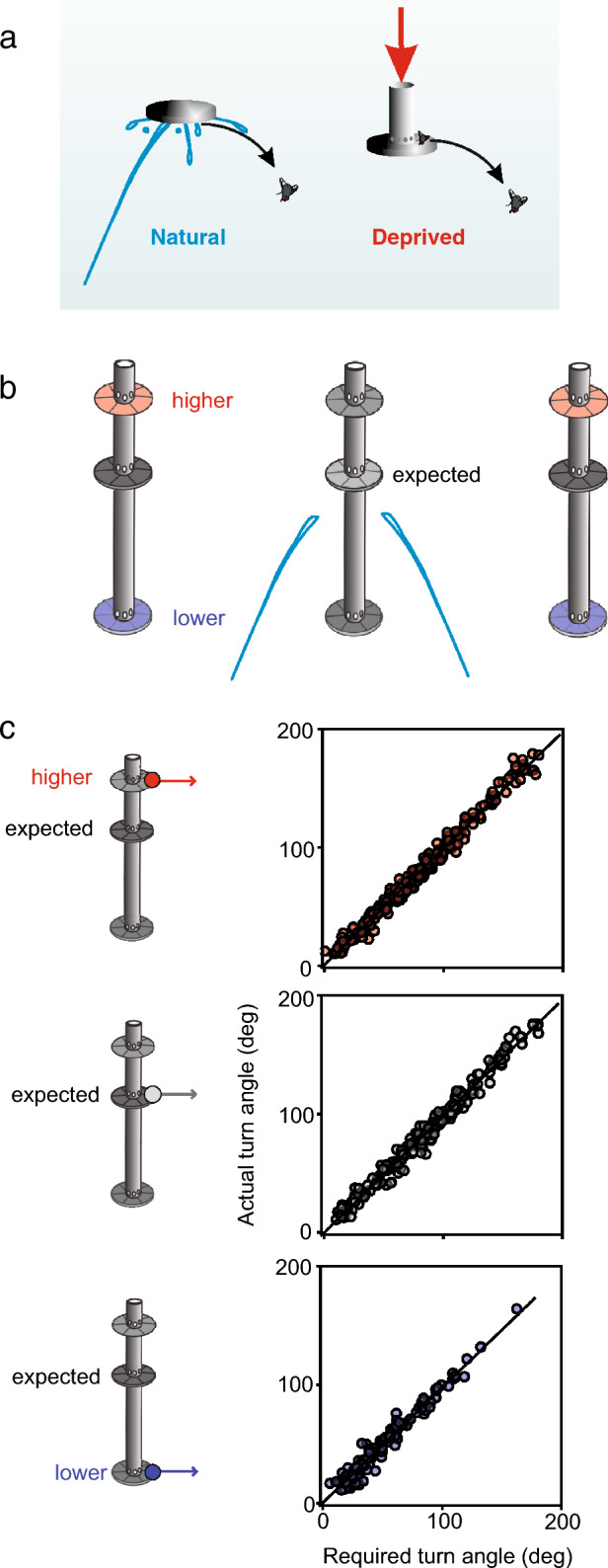
The decision is only driven by information sampled after onset of prey movement. **a** An experiment to show that no *a priori* cues are used in the predictive start decision. The accuracy and latency of predictive starts were compared when a group of archerfish had dislodged prey with their shots (‘Natural’) or when the fish had no control over timing, speed, and direction of prey movement (‘Deprived’). In the ‘Deprived’ condition, prey is on the top side of a non-transparent disk and set into motion by an airflow, in the ‘Natural’ condition it is located on the bottom of the disk. Although much useful information would be available in the ‘Natural’ condition, start decisions were made equally fast and with equal accuracy. **b** An experiment to show that also initial height is determined when prey falls, not before. In this experiment, a group of archerfish were shooting at one (‘expected’) of nine platforms and food came from this expected platform. As they fired to this platform occasionally prey was dislodged from higher or lower platforms located to the left or to the right. **c** Evaluation of the aims taken in the C-start decisions when height of actual prey motion was as expected (gray), higher (red) or lower (blue). Turns actually made are plotted against turns required for perfect alignment in each of the recorded responses. Distribution of errors (not shown) to real landing point are not significantly different but errors to the landing points that would be inferred based on the expected height level were highly significantly different both in the ‘higher’ and ‘lower’ condition and same conclusion also holds when the speed set by the C-starts is analyzed (see Reinel and Schuster 2018a). Adapted from Schlegel and Schuster (2008) and Reinel and Schuster (2018a)

Perhaps one of the most intriguing aspects of the predictive start decisions is that they are only driven by information sampled during the initial movement of dislodged prey but not by information that is demonstrably already available somewhere in the brain of the responding fish. In a natural hunting situation, the shooter and other bystander archerfish would normally have useful information for their decisions long before their prey starts to fall. They know where the prey is located and at least the shooter must also know the prey’s height above the water surface to adjust its jet accordingly (Gerullis and Schuster 2014). The shot itself also would deliver useful cues (visual and acoustic) to alert everyone that prey movement is now to be expected. Furthermore, looking from where the shot came and how it is going to impact could allow at least some estimate of the most likely directions in which prey will go off. Interestingly, a simple experiment (Fig. 4a) suggested that none of this information is fed into the decision-circuitry. Rather this circuitry somehow seems to be bound to use independent information, gathered after the onset of prey movement. In this experiment, accuracy and latency of the predictive C-starts of a group of archerfish were compared in two situations: First, when the fish would have all potential *a priori* cues. In the second situation, however, the fish could not see their prey and could not know when it will start to move, which initial speed it will have and in which direction it will go off. Specifically, there was no trigger signal alerting the fish when to expect movement. This is because prey movement was now commanded by the experimenter. Nonetheless, latency and accuracy of the predictive starts came out just the same in both conditions (Schlegel and Schuster 2008).

These experiments were extended further to show that also no prior knowledge about position and height is needed (Fig. 4b). In experiments with several platforms, it is possible to make the fish look and shoot at only one platform by placing food on it and by releasing food only from this platform. Then, prey is sometimes launched from the other platforms that are displaced horizontally and/or vertically (Schlegel and Schuster 2008; Reinel and Schuster [Bibr CR37][Bibr CR30], b). When the predictive starts were later compared that were either made when prey came from where the fish should have expected it or from a different position and height, it became clear that prior knowledge was not necessary (Reinel and Schuster 2018a). By analyzing aim (Fig. 4c) and speed (see Reinel and Schuster 2018a) it was possible to determine if the fish aimed according to expected or according to actual height. The fish clearly set their starts based on position and height as determined after onset of prey motion but not based on potentially expected values. Presently, it is not known what allows the fish to determine distance so quickly, but neither binocular processing nor accommodation appears to be used. A series of experiments with agar-coated flies suggested that the fish do not use a set focus distance (e.g., the ‘expected’ distance) to use the degree of image blurring as a cue to distance (Reinel and Schuster 2018b). An idea that would be compatible with these and other (Reinel and Schuster 2016) results would be that height is determined from the rate of looming. So, while meaningful information about height would be available in a natural context, this information is demonstrably not used in the predictive start decision. Rather, the apparently difficult route is taken to determine distance independently during the very short interval of about 40 ms that follows the onset of falling motion of prey.

## The decision relies on some form of representation of how objects fall

The finding that the decision uses only information that is gathered during the initial phase of falling is the basis for a variety of experimental approaches. For instance, it allows to test archerfish with randomly assigned combinations of the initial values of prey motion—speed, direction, initial height—that determine where prey will land. So, it is possible to have the fish face one specific combination of speed, height, and direction of prey movement in one test and another unpredictable assembly of initial values in the next test and so on. To see how this works, consider the results shown in Fig. 4c. Here in one test, the fish would, for instance, face a fly going off from the expected height and with such direction and speed as to require the fish to turn by a certain angle. In the subsequent test, however, the fish may face a fly that falls from another height, at another speed and into another direction, requiring the fish to make a different turn. All turns shown in Fig. 4c are recorded in experiments in which the fish were challenged with such random combinations of initial height, speed and direction of prey and had to select starts as appropriate (in aim and speed) for their own position and orientation. So, although the fish did never know which combination to expect, the responses were accurate regardless of the size of turn the fish had to make but also regardless from the distances the fish had to cover in the remaining time (see Reinel and Schuster 2018a). To further appreciate the findings, it is important to know that the predictive starts do not rely on *a priori* knowledge, for instance of the size of the moving prey or of its distance from a structured background. This can be tested by occasionally presenting prey of unusual size or with unusual distance from a structured background (e.g., Reinel and Schuster 2018b). Moreover, accuracy of setting speed and aim were always determined only for the first responding fish of the group so that it could only be guided by the falling motion.

The ability of the fish to respond to any combination of initial motion values of falling prey and for all possible initial orientations and positions requires the fish to use some kind of ‘representation’ of how prey falls. In principle, such a representation could consist of a look-up table in which the fish stores, after each successful predictive C-start, the turn size and speed it had to select given its initial position and orientation as well as given the perceived prey movement. As simple as this might seem, using such a look-up table would be rather demanding, given the enormous size it would have to have. A much more efficient way would be to represent the underlying rule that connects all variables. In the case of small prey, that typically would be completely drenched by the shot, this would just be the laws of ballistics without air friction (Rossel et al. 2002; Reinel and Schuster 2018a).

## Functional stability: keeping the starts accurate under fluctuating environmental conditions

Because archerfish operate close to the water surface, patrolling and looking for potential prey, they are easily spotted from above in the wild. While this might not be problematic, their visibility increases dramatically as soon as they launch a predictive C-start. The water surface waves created in this maneuver are visible from far away and could easily attract birds like herons or kingfishers (that are common where archerfish are found), resulting in an increased predation risk. Additionally, the energetic costs for launching the powerful predictive C-start can probably not be neglected. Predatory and energetic costs make it advisable that starts should only be launched when they are sufficiently accurate. However, in their mangrove habitats, light levels and contrasts at which falling prey can be seen vary as the fish move between various spots. Also, water temperature, a parameter that affects vision, neuronal processing, and muscle contraction and that is known to strongly affect C-starts, fluctuates as the fish move from one spot to the next. This raises the question of how the archerfish predictive start decisions are affected by the fluctuating conditions they do face in the wild. Can the decisions somehow maintain stable functionality, i.e., can the fish produce accurate C-starts in fluctuating environments as would seem required to optimally secure their prey?

**Fig. 5:**
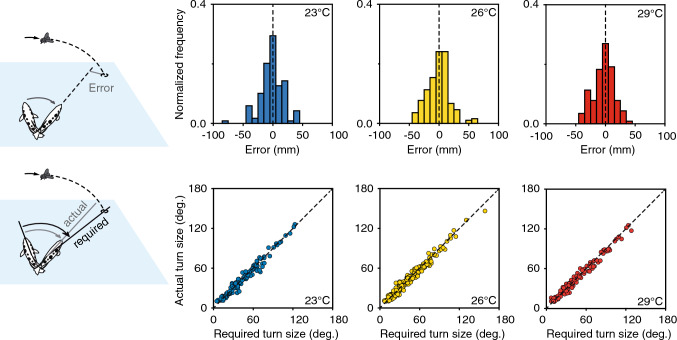
Functional stability in the predictive C-start decisions. In the wild, hunting archerfish face rapid fluctuations in conditions that influence processing and motor output relevant for their C-starts and yet the accuracy of their start decisions appears to be buffered against such changes. Example to show how accuracy remains constant when temperature changes. Upper row shows distribution of errors made at three temperatures. Diagrams on the left illustrate how error to later landing point is determined right at the end of the C-start. Lower row shows the turns that were made in each response and the turns that would have been required for perfect alignment to the later landing point. This shows that at all temperatures, responses were made in a wide angular range and were equally accurate. Adapted from Krupczynski and Schuster (2013)

Interestingly, one consistent finding so far is that once launched, archerfish predictive starts are equally accurate under all conditions. In some cases, however, the likelihood changes at which falling prey elicits a C-start response. For example, at lower light levels and with low visual contrast, the same type of falling motion elicits fewer C-starts and below a certain light level no C-starts are produced although the fish are demonstrably still motivated to hunt, as can be conveniently assayed by their shooting behavior. However, all predictive C-starts that are triggered, regardless of at what light level they were initiated, are of equal average accuracy (Schlegel and Schuster 2008). A similar situation holds after changes in temperature (Fig. 5). In cooler water, the latency of the predictive starts increases as does the duration of the C-start maneuver. Nevertheless, all C-starts that are produced are of equal accuracy and follow the same error distribution, no matter at which temperature they were produced (Krupczynski and Schuster 2013). Interestingly, even the likelihood to launch a predictive start decision remained constant within the temperature range that would be relevant for hunting in the wild.

## Comparing archerfish high-speed decisions with the standard model of decision-making

The study of decision-making has largely been influenced by findings on saccadic decisions made by monkeys in a visual forced choice task with two alternatives (e.g., Gold and Shadlen 2007; Churchland et al. 2008; Hanks et al. 2014). In the experiments, a monkey views a screen from fixed distance and orientation and sees an assembly of dots, each of which can move either to the left or to the right. The monkey’s task is to decide if more dots move to the left or to the right. The task is made harder by decreasing the percentage of dots moving in one direction from 100 to 50%. From these studies, it has become clear that (1) the decisions become less accurate when they must be made in lesser time, (2) when the animal is free to refuse cooperating in the task it will do so when accuracy is low, and (3) raising the number of options from two to four decreases the accuracy of the decisions (Churchland et al. 2008). The findings not only capture many aspects of decision-making but could be explained simply by recordings in the lateral intraparietal cortex. With increasing evidence in favor of each of the two options, two sets of neurons, one for each option, increased their firing rate until one of them first reaches a threshold. The success of this model should, however, not make us forget that there are decisions that simply do not offer the time for such a solution and yet need to be complex and accurate. The archerfish’s predictive C-start decision is a good example for this. It fails to obey any of the characteristics (1)–(3) noted above: Accuracy is unrelated both to latency and to the probability that the fish will respond with a predictive start. Furthermore, even adding a new variable (adding many more than two additional options) did not affect latency or accuracy (Reinel and Schuster 2016). This is not because the predictive start decisions are simple and made between a limited number of pre-set options (Wang 2008). To the contrary, these decisions are made in a continuum of four variables: vertical initial speed, horizontal initial speed, azimuthal direction, and initial height. They are not made from a fixed vantage point but allow the fish to achieve accuracy in speed and turn angle over the full range of orientations and from all relevant distances. It should be stimulating to understand why some complex decisions (complex by the large number of options) apparently are not bound by the type of constraints that are seen in the saccadic decisions of monkeys. In rapid decisions, such as the archerfish’s start decisions, other mechanisms must have evolved to combine utmost speed with accuracy.

### Are the archerfish fast-start decisions initiated by the Mauthner neuron?

**Fig. 6:**
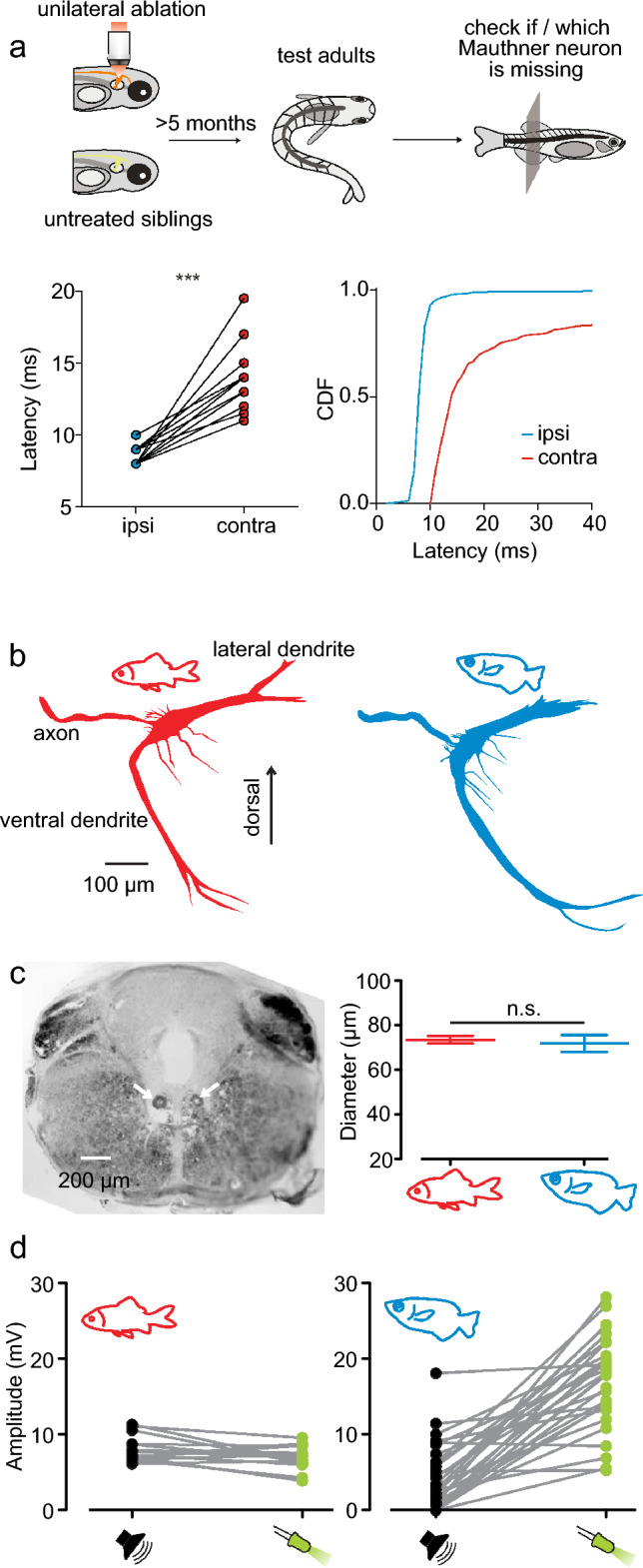
Evidence used in the discussion of an involvement of the archerfish Mauthner neuron (MN) in triggering archerfish C-starts. **a** The importance of the MN in adult fish. Larval zebrafish had either their right or left MN (including its axon) ablated as confirmed using two-photon microscopy. They were subsequently raised together with untreated siblings. At least 5 months later C-starts were elicited by a standardized stimulus that caused the fish to bend to the left or to right side. After accumulating enough starts to both sides, each experimental fish was sacrificed to determine whether the left or the right Mauthner axon was missing (or none). Diagrams show the findings for the unilaterally ablated fish and demonstrate that, in the same individuals (lines in left diagram), latency was always shorted in C-starts that could recruit the remaining Mauthner axon (ipsi) than in the starts that could not (contra). CDF = cumulative distribution function. **b** Intracellular filling of the MN in equally sized goldfish (red) and archerfish (blue) revealed no major differences. See Machnik et al. (2018a) for detailed measurements. **c** Left: The axons of the archerfish MN are by far the largest in the spinal cord, but their diameter is not significantly different from that in goldfish of similar total length (right). **d** The only difference found in a detailed comparative analysis was that in archerfish postsynaptic potentials (PSPs) elicited by acoustic stimuli and light flashes, whose intensities were chosen to elicit PSPs of comparable size in goldfish, were larger for the visual stimuli. Adapted from Hecker et al. (2020a), Machnik et al. (2018a, b)

To quickly bend into the shape of a letter ‘C’, it is essential that all trunk muscles contract on only one side, and for maximum force all muscle fibers should contract simultaneously. This can be achieved by sending the command down a thick and fast-conducting axon. The Mauthner neuron is ideally suited for just this task (e.g., Korn and Faber 2005; Sillar 2009; Sillar et al. 2016). Each axon of the two Mauthner neurons crosses the fish’s midline and runs down the spinal cord. The two neurons are wired such that only one of them fires one and only one action potential. So, if the Mauthner neuron located on the left side of the fish gets activated, it sends an action potential down the right side of the fish (because the axon crosses the midline), whose rapid spreading causes almost simultaneous contraction of trunk muscles on the right side, thus powerfully bending the fish towards the right. As simple as this view is it has met with remarkably fierce resistance. First, ablation experiments have repeatedly found at least some equally short-latency, high-power C-starts (e.g. DiDomenico et al. 1988; Eaton et al. 1982, 1991, 1995; Gahtan and Baier 2004; Lacoste et al. 2015; Liu and Fetcho 1999; Zottoli et al. 1999). Second, it has been argued that a few milliseconds of increase in latency would not be of any consequence for survival (DiDomenico et al. 1988; DiDomenico and Eaton 1988; Eaton et al. 1991, 1995). Moreover, even an unusually low capacity to regenerate was described particularly for the large Mauthner axon (Bhatt et al. 2004), making it even more cumbersome to assume why this neuron should be of particular relevance for triggering live-saving C-starts. The finding that low-latency high-power C-starts should still be possible when the Mauthner cells are lost does fit the generally accepted idea that no single neuron can underlie important behavioral functions (the textbook ‘grandmother neuron doctrine’). However, it makes it puzzling, why then these large neurons are still present, when their function can, at least in principle, be taken over by smaller neurons. It recently turned out, that completely unilaterally removing specifically one Mauthner neuron, including its axon, specifically removed the ability of zebrafish larvae to produce short-latency high-power C-starts to the side in which the axon was missing but not to the side in which it was still present (Hecker et al. 2020a). In these experiments, the complete and slow Wallerian degeneration of the axon was followed in a two-photon microscope until the complete axon was absent. Stimuli were given at various stages of the axon degeneration to probe C-starts. These stimuli were selected for their ability to elicit activity in all neurons of the so-called Mauthner series (e.g., Liu and Fetcho 1999), so that other neurons could in principle substitute for the absent Mauthner neuron. However, removal of the Mauthner axon completely removed all short-latency, high-power C-starts. This finding also allowed what appears to be the first direct experimental test of the debated issue of the actual survival value of these maneuvers. In tests caried out with a natural predator, sham-ablated larvae with intact Mauthner cells had a higher chance of surviving the predator attacks (Hecker et al. 2020a). Furthermore, raising unilaterally ablated larvae and examining them double-blind months later as adults showed that this deficiency is never restored even during the massive transformation from the larva to an adult fish (Hecker et al. 2020a). In other words, the Mauthner neuron is essential for driving powerful short-latency C-starts both in larval and adult fish and removing it does have clear consequences (Fig. 6a). It also turned out that—in line with the unique importance of this neuron—the regenerative capability of the Mauthner axon is not generally low but instead strongly dependent on where on its length the axon is lesioned. In the front end of the fish, where the effect of losing the axon on C-start latency is expected to be greatest, regeneration is impressively rapid (Hecker et al. 2020b).

Archerfish do have Mauthner neurons (Fig. 6b) that have a so-called axon cap, which means that they can be found in the hindbrain by an electrical signature, the same way as the goldfish Mauthner cell is found (Machnik et al. 2018a, b). The archerfish Mauthner axon has the largest diameter of all axons that run down the archerfish spinal cord (Fig. 6c). Both morphologically and functionally the archerfish Mauthner neurons are like those of the goldfish with the only major difference being their higher visual sensitivity. So, the key question now is whether archerfish can use an apparently ‘standard’ Mauthner neuron for something as sophisticated as their predictive C-start decisions. An easily acceptable view would seem that archerfish use this neuron for escapes but not for their predictive starts. But why then are the escape C-starts and the predictive C-starts not easily distinguishable from their kinematics? Is there perhaps some other circuitry that achieves the same top-performance as the escape C-start? If that was the case, then, again, the use of other (smaller) neurons would allow to produce top-performance C-starts so that similar networks of smaller cells with thinner axons could also be used to produce top-performance escape C-starts, making the archerfish Mauthner neuron obsolete. The simplest explanation, also considering the findings in adult zebrafish (Fig. 6a), is that the archerfish C-starts, escapes and predictive starts alike, are initiated by an action potential that travels down the thick axon of one of the Mauthner neurons. Clearly, a remarkable and highly efficient preprocessing is needed, and this will probably differ among escapes and predictive starts, but the final ‘go’ will have to be given by firing one of the two Mauthner neurons. The archerfish predictive C-starts, thus, lead to the intriguing question of what can and cannot be done with an individual heavily compartmentalized (e.g., Korn and Faber 2005) identified neuron in a vertebrate brain.

## Conclusion

It is often assumed that precise decisions cannot be fast and fast decisions can either not be precise or must be made between a few pre-set options. A look at the predictive C-start decisions of archerfish shows that this view cannot generally be true. The context in which these fish make their high-speed decisions demands speed and accuracy to be combined and maintained over a wide operating range—otherwise, their decisions would be useless.
